# Dispersed repeats and inverted repeat expansion drive major plastomic rearrangements in *Calliandra haematocephala* (Leguminosae: Mimoseae)

**DOI:** 10.3389/fpls.2025.1673127

**Published:** 2025-10-03

**Authors:** Libo Zhao, Kailun An, Wei Gu, Qing Lu, Ding-Jie Wang, Zuoying XiaHou, Rong Zhang, Ting-Shuang Yi

**Affiliations:** ^1^ The Germplasm Bank of Wild Species and Yunnan Key Laboratory of Crop Wild Relatives Omics, Kunming Institute of Botany, Chinese Academy of Sciences, Kunming, China; ^2^ University of Chinese Academy of Sciences, Beijing, China

**Keywords:** plastome evolution, inverted repeat expansion, rearrangement, nucleotide diversity, positive selection

## Abstract

Structural variations in legume plastomes impact phylogenetic and evolutionary studies. In this study, we focus on the tribe Mimoseae by integrating a newly assembled plastome of *Calliandra haematocephala* (from PacBio sequencing data) with 15 previously published plastomes representing major lineages, to analyze structural rearrangements, repeat sequences, and selection pressures. The plastome of *C. haematocephala* revealed extensive structural rearrangements and a *ca*. 14-kb expansion of the inverted repeats (IRs) into the large single copy (LSC) region, resulting in IRs of 42,069 bp. It also contained a high abundance of clustered dispersed repeats (> 90 bp). These features potentially contribute to significant plastome rearrangements, making it the largest plastome (200,623 bp) recorded to date in Mimoseae and, more broadly, in Leguminosae. Nucleotide diversity (Pi) analysis identified several highly variable regions (Pi > 0.03), including the genes *accD*, *rps18*, *clpP*, and multiple non-coding loci, suggesting their potential as molecular markers. Selection pressure analyses detected positive selection (*dN/dS* > 1) in *clpP*, *ycf2*, and *rps17*, suggesting possible roles in adaptive evolution. Branch-specific positive selection was also found in genes such as *rpoC1* and *atpA* within the Calliandra clade, indicating lineage-specific adaptive pressures. This study highlights the dynamic evolution of plastomes in Mimoseae and offers new insights into their structural diversity and adaptive evolution.

## 1 Introduction

The plastome is a circular molecule typically exhibiting a conserved quadripartite structure in most autotrophic plants: two inverted repeats (IRs) separate the large single copy (LSC) and the small single copy (SSC) regions (Jansen and Ruhlman, 2012; Wang et al., 2024). Plastome size generally ranges from 120 to 160 kb (Bock, 2007), though frequently influenced by IR expansion/contraction or loss (Jansen and Ruhlman, 2012). Extensive IR expansion occurs in plastomes of multiple taxa such as *Pelargonium* L’Hér. ex Aiton (Geraniaceae), where IRs exceed 87 kb, yielding plastomes > 242 kb (Chumley et al., 2006; Weng et al., 2017). IR contraction is also common, e.g., plastomes of *Pentasachme caudata* Wall. ex Wight (Apocynaceae) (Wang et al., 2023) and *Dicorynia paraensis* Benth. (Fabaceae) (Bai et al., 2020). Some plastomes of non-autotrophic plants lose one IR copy (Huang et al., 2011), concomitant with the loss of photosynthesis-related genes (Krause and Scharff, 2014), resulting in significant plastomic variations. IR loss also occurs in autotrophic seed plants, such as the Inverted Repeat-Lacking Clade (IRLC) and *Camoensia* Welw. ex Benth. of Fabaceae (Choi et al., 2019; Cauz-Santos et al., 2020), certain species in Geraniaceae (Guisinger et al., 2011; Blazier et al., 2016; Ruhlman et al., 2017), and some species of Cactaceae (Sanderson et al., 2015). These plastomes usually exhibit considerable plastomic changes. Some IR-retaining species also exhibit substantial plastome structural variations, such as those in certain species of Campanulaceae (Wu and Chaw, 2014), Oleaceae (Cosner et al., 2004), Plantaginaceae (Zhu et al., 2016), and *Pelargonium* (Lee et al., 2007). Plastome size and structural variation are influenced not only by IR dynamics but also by repetitive sequences, including tandem repeats (Jo et al., 2011; Dugas et al., 2015) and dispersed repeats (Cosner et al., 1997; Haberle et al., 2008; Jansen and Ruhlman, 2012).

Significant plastome structural variation occurs among legumes, particularly in subfamily Papilionoideae. A 50-kb inversion in the LSC region characterizes plastomes of most papilionoids, except a few early-diverged lineages (Doyle et al., 1996; Chen et al., 2025). Plastomes of *Vigna radiata* (L.) R.Wilczek and *Phaseolus vulgaris* L. exhibit an additional 78-kb inversion (Palmer et al., 1988), while an additional 36-kb inversion within the 50-kb segment is observed in *Lupinus luteus* L. (Martin et al., 2014). IRLC plastomes lack complete IRs (Palmer et al., 1987; Doyle et al., 1996; Wojciechowski et al., 2000), with many species exhibiting significant plastome structural rearrangements like gene/intron losses (e.g., *Cicer arietinum* L.) (Jansen et al., 2008). In addition to the above-mentioned two subfamilies, a 7.5-kb inversion was found in *Tylosema esculentum* (Burch.) A.Schreib. (Cercidoideae), and this structural variation was initially proposed to occur throughout *Bauhinia* s.l. (Kim and Cullis, 2017), but subsequent studies demonstrated that it is restricted to *Tylosema* (Schweinf.) Torre & Hillc. (Wang et al., 2017b).

Mimosoideae, redefined as tribe Mimoseae (subfamily Caesalpinioideae), comprises about 100 genera and 3500 species distributed in pantropical regions (Ringelberg et al., 2022; Bruneau et al., 2024). Many species are ecologically dominant in major tropical biomes (Lewis et al., 2005; LPWG, 2013). Some species are of high economic value as fodder crops [*Leucaena leucocephala* (Lam.) de Wit], ornamental plants (species of *Albizia* Durazz. and *Calliandra* Benth.), timber trees (species of *Acacia* Mill., *Anadenanthera* Speg., and *Prosopis* L.), and sources of food thickeners (species of *Acacia*) (Lewis et al., 2005). Plastomes of this tribe exhibit a relatively conserved structure. However, plastomes of a clade formed by tribe Ingeae and *Acacia* s.s. contain a *ca*. 13-kb IR expansion into the SSC (Dugas et al., 2015; Williams et al., 2015), representing the largest known legume plastomes (from 174,217 bp to 178,887 bp) (Wang et al., 2017a). This clade, designated as the Inverted Repeat-expanding clade (IREC) (Wang, 2017), exhibits additional structural variations such as IR-LSC junction alterations, intron losses, gene duplications, and inversions (Wang et al., 2017a). Our preliminary work revealed a large, highly variable plastome in the ornamental species *Calliandra haematocephala* Hassk. Given that *Calliandra* resides within the tribe Mimoseae (Bruneau et al., 2024) and its plastome variations remain uncharacterized, it presents an excellent opportunity for making an investigation.

In this study, we selected 15 species from representative genera across the major clades and grades within Mimoseae to capture the extent of the plastomic structural diversity. We investigated structural divergence by comparing the newly sequenced plastome of *C. haematocephala* with those of these 15 representative plastomes of this tribe. We conducted a comprehensive phylogenetic analysis using 16 plastomes to clarify the relationship within Mimoseae. The objectives of this study are: 1. To elucidate the unique plastome structural variations of the *C. haematocephala* plastome; 2. To identify hypervariable regions that may serve as informative molecular markers for future phylogenetic studies and species identifying in Mimoseae. We report the plastome of *C. haematocephala*, revealing extensive rearrangements despite IR retention. This resource facilitates further exploration of plastome structural dynamics in Mimoseae.

## 2 Materials and methods

### 2.1 Sampling, genomic DNA extraction, and sequencing

Our study incorporated 15 assembled plastomes from GenBank and one newly sequenced plastome, representing 16 clades or grades of the tribe Mimoseae (**Supplementary Table S1**). The 15 selected species, each representing a genus from the major clades or grades of Mimoseae, reflect the structural diversity of plastomes in this tribe. We selected *Adenanthera microsperma* Teijsm. & Binn., an early-divergent species of Mimoseae (Bruneau et al., 2024), as the outgroup for the taxa of interest; its plastome was retrieved from GenBank (**Supplementary Table S1**). Fresh leaf tissue of *C. haematocephala* was flash-frozen in Liquid nitrogen collected from Xishuangbanna Tropical Botanical Garden, Chinese Academy of Sciences (Menglun Town, Xishuangbanna Dai Autonomous Prefecture, Yunnan Province, China; voucher specimen deposited in the Kunming Institute of Botany, Chinese Academy of Sciences). Samples were subsequently sent to BIOMARKER Technologies Co., Ltd. (Beijing) for total DNA and RNA extraction. Target-enriched fragment selection was performed using BluePippin, followed by library preparation according to PacBio standard protocols. The sequencing libraries were ultimately sequenced for full-length sequencing on a PacBio Sequel II platform.

### 2.2 Assembly and annotation of plastomes

Oatk v1.0 (Zhou et al., 2024) and TIPPo v2.3 (Xian et al., 2025) were used to assemble the complete plastome of *C. haematocephala* from raw sequencing data, with assemblies cross-validated to ensure accuracy. All 17 plastomes (including the outgroup) were annotated using PGA (Qu et al., 2019; Zhang et al., 2025). Finally, manual adjustments of the annotation were performed using Geneious v.9.0.2 (Kearse et al., 2012). Physical maps were generated using the online tool OGDRAW (Greiner et al., 2019). Genome collinearity was analyzed with the progressiveMauve (Darling et al., 2004) plugin in Geneious (Kearse et al., 2012).

### 2.3 Phylogenetic analysis

Eighty protein-coding genes (PCGs) were extracted from 17 plastomes of Mimoseae, aligned using MAFFT v7.487 (Katoh et al., 2002), and concatenated into a data matrix. Concurrently, a whole plastome alignment (designated as Full-Con) was constructed using MAFFT and PhyloSuite v1.2.2 (Zhang et al., 2020). Maximum likelihood (ML) trees were inferred from both matrices using RAxML v8.2.12 (Stamatakis, 2014) under the GTRGAMMA model with 1000 rapid bootstrap replicates.

### 2.4 IR boundary analysis and nucleotide diversity

To characterize IR expansion/contraction across 16 plastomes from Mimoseae (excluding the outgroup), boundaries of IR/SC were visualized and compared using IRscope (Amiryousefi et al., 2018). Whole plastomes were aligned under the shuffle-LAGAN model and visualized using mVISTA (Frazer et al., 2004). Sixty-three PCGs and 86 non-coding regions including introns or intergenic spacers with a length exceeding 200 bp were aligned using MAFFT; nucleotide diversity (Pi) was subsequently calculated via sliding window analysis using DnaSP v. 6.10 (Rozas et al., 2017) with a window length of 600 bp and a step size of 200 bp.

### 2.5 Repeat sequences identification and codon usage analysis

Simple sequence repeats (SSRs) were identified using the online software MISA (Beier et al., 2017) (https://webblast.ipk-gatersleben.de/misa/) with minimum thresholds of 10, 5, 4, 3, 3, 3 for mono-, di-, tri-, tetra-, penta-, and hexanucleotides, respectively. Long repeat sequences (forward, palindromic, reverse, and complement repeats) were detected with REPuter (Kurtz and Schleiermacher, 1999; Kurtz et al., 2001) (https://bibiserv.cebitec.uni-bielefeld.de/reputer/) using the following parameters: minimum repeat length = 30, Hamming distance = 3, minimum identity = 90%, and maximum computed repeats = 150.

### 2.6 Selection pressure analyses

The alignment of PCGs was generated using the MAFFT plugin with “Codon” mode, followed by format conversion to PML using the “Convert Sequence Format” tool in PhyloSuite v1.2.2 (Zhang et al., 2020). For phylogenetic reconstruction, sequences from all 16 species of Mimoseae were aligned using MAFFT v7.487 (Katoh et al., 2002) with default parameters. ML trees of these PCGs alignments were inferred respectively using RAxML v8.2.12 (Stamatakis, 2014) under the GTRGAMMA substitution model and 1000 rapid bootstrap replicates.

Non-synonymous/synonymous rate ratios (*dN/dS*) were calculated using CodeML in PAML v4.10.7 (Yang, 2007) with the ML tree and PML-formatted alignment files as inputs. Branch model analyses were employed to assess selective pressures. Comparative analyses between the one-ratio and the two-ratio models were performed to identify variation in selective pressure across branches in PCGs. Model comparisons were executed through likelihood ratio tests (LRT) (Yang et al., 2005), where designated clades were specified as foreground branches and remaining lineages as background. CodeML was run under branch models (runmode = 0, model = 0 or 2, NSsites = 0) as described in the PAML manual (Álvarez-Carretero et al., 2023). CodeML configuration files were established in accordance with official documentation (Álvarez-Carretero et al., 2023), applying this protocol to all tested clades (Lu et al., 2024).

## 3 Result

### 3.1 Plastome size and features

**Figure 1:**
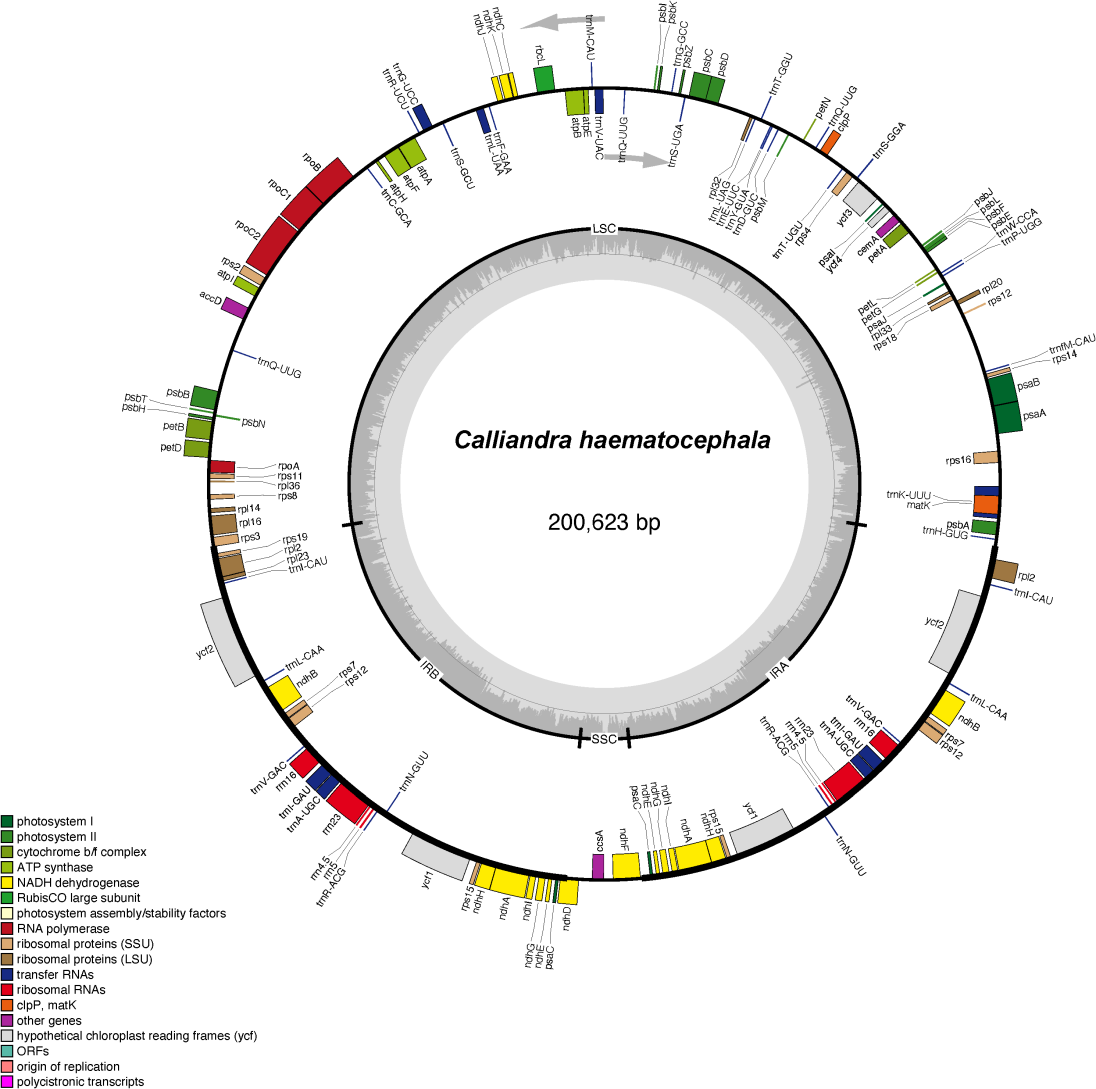
The circular plastome map of *Calliandra haematocephala*. Genes inside the circle are transcribed clockwise, while genes outside the circle are transcribed counterclockwise. Genes belonging to different functional groups are marked with different colors. The darker and lighter gray areas in the inner circle corresponds to the GC content and AT content, respectively.

We assembled and annotated the newly sequenced plastome of *C. haematocephala* and re-annotated 15 additional plastomes of Mimoseae from GenBank. All plastomes exhibited a circular, quadripartite structure comprising the LSC region, SSC region, and IR regions. Total lengths ranged from 159,963 to 200,623 bp, with the LSC regions ranging from 87,462 to 110,424 bp, SSC regions ranging from 4,470 to 19,392 bp, and IR regions ranging from 25,341 to 42,069 bp. The total GC content of these plastomes ranged from 35.0% to 36.6%. The plastome size of *C. haematocephala* is 200,623 bp, with an LSC length of 110,424 bp, an SSC length of 6,061 bp, and a single IR length of 42,069 bp. The structure of the plastome of *C. haematocephala* is illustrated in **Figure 1**.

**Figure 2:**
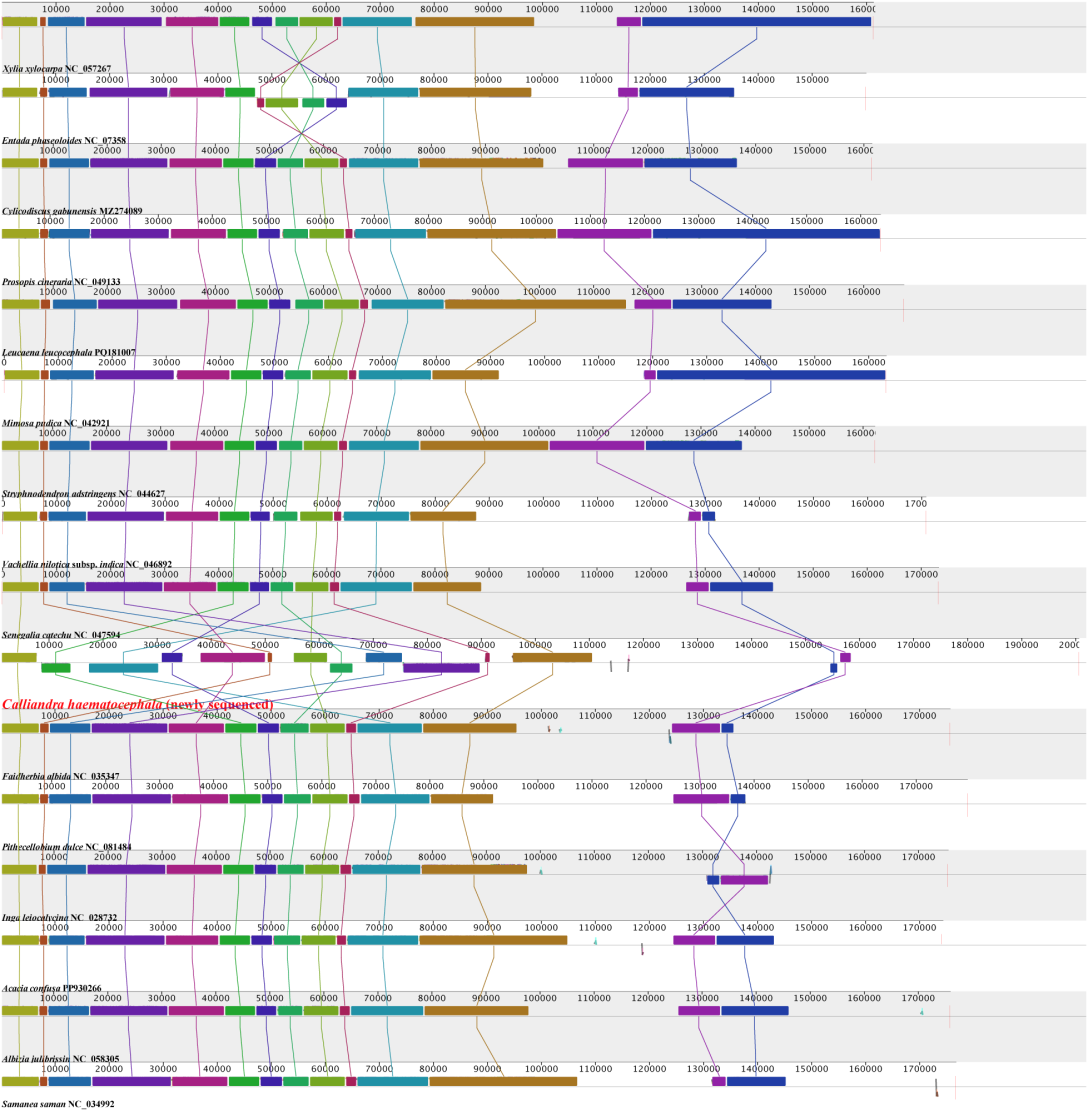
Collinearity comparison analysis of 16 plastomes from tribe Mimoseae. The horizontally arranged colored rectangles linked by sane colored line represent locally collinear blocks, indicating homologous regions with conserved gene order and sequence similarity.

The 16 plastomes of Mimoseae exhibited relatively high gene content and collinearity (**Figure 2**). Fifteen plastomes maintained nearly identical gene arrangements, while the plastome of *C. haematocephala* showed substantial structural variation. Annotation revealed that these 15 plastomes contained 128 to 142 genes, including 83 to 95 protein-coding genes, 37 tRNA genes, and 8 rRNA genes. In contrast, *C. haematocephala* contained 137 genes, including 90 protein-coding genes (15 in IR regions), 39 tRNA genes (6 in IR regions), and 8 rRNA genes (4 in IR regions).

*Entada phaseoloides* (L.) Merr. exhibited a rearrangement distance of 1, corresponding to a single inversion event involving four contiguous blocks. *Inga leiocalycina* Benth. showed a rearrangement distance of 1, associated with a single inversion of two contiguous blocks. *Calliandra haematocephala* displayed a rearrangement distance of 12, reflecting extensive genomic rearrangement involving multiple translocations and inversions (**Figure 2**; **Supplementary Table S2**).

### 3.2 IR expansion and contraction in 16 plastomes of Mimoseae

Among the 16 analyzed plastomes of Mimoseae, only plastomes of four species [*Cylicodiscus gabunensis* Harms, *L. leucocephala*, *Prosopis cineraria* (L.) Druce, and *Stryphnodendron adstringens* (Mart.) Coville] exhibited canonical IRs without significant expansion or contraction, with IR lengths ranging from 25,931 bp (*P. cineraria*) to 26,062 bp (*S. adstringens*). Nine species [*Acacia confusa* Merr., *Albizia julibrissin* Durazz., *C. haematocephala*, *Faidherbia albida* (Delile) A.Chev., *I. leiocalycina*, *Pithecellobium dulce* (Roxb.) Benth., *Samanea saman* (Jacq.) Merr., *Senegalia catechu* (L.f.) P.J.H.Hurter & Mabb., and *Vachellia nilotica* subsp. *indica* (Benth.) Kyal. & Boatwr.] exhibited substantial IR expansions of approximately 13–16 kb into the SSC region, resulting in IR lengths ranging from 39,347 bp (*V. nilotica* subsp. *indica*) to 42,069 bp (*C. haematocephala*).

Except for *C. haematocephala*, whose IRB/SSC junction (JSB) was located within *ndhD*, duplicating only eight complete protein-coding genes (from *ycf1* to *psaC*), the IRs of the remaining eight species contained nine complete protein-coding genes (from *ycf1* to *ndhD*). In *A. confusa*, *A. julibrissin*, *F. albida*, *P. dulce*, *S. saman*, and *S. catechu*, the JSB shifted into *ndhF*, duplicating its 3’ end (19–205 bp), while the SSC/IRA junction (JSA) relocated from *ycf1* to between *ccsA* and *ndhD* (except *F. albida* having JSA within the stop codon of *ccsA*).

In *C. haematocephala*, the JSB shifted into *ndhD*, duplicating its 5’ end (538 bp), and the JSA relocated from *ycf1* to between *ndhF* and *psaC*. In *I. leiocalycina*, JSB occurred between *ndhD* and *ccsA*, while JSA shifted into *ndhF*, duplicating its 3’ end (6 bp). In *V. nilotic*a subsp. *indica*, JSB relocated between *ndhD* and *ndhF*, and JSA shifted to between *ccsA* and *ndhD*.

Four species (*A. julibrissin*, *F. albida*, *I. leiocalycina*, and *S. saman*) exhibited LSC/IRB junctions (JLB) within *rps19*, duplicating its 5’ end (100–105 bp). *Calliandra haematocephala* displayed an additional 0.7-kb IR expansion into LSC, shifting its JLB into *rps3* and incorporating the entire *rps19* and 31 bp of *rps3*. In *P. dulce*, a 1.7-kb IR expansion into the LSC positioned JLB between *rps3* and *rpl16*, adding *rps3* and *rps19* to IR. *S. catechu* and *V. nilotica* subsp. *indica* showed a 1.2-kb IR expansion into LSC, relocating JLB between *rps19* and *rpl23* and incorporating *rps19*. Conversely, *A. confusa* exhibited a 0.3-kb IR contraction, shifting its JLB into *rpl2* and transferring the entire *rpl2* (4 bp) to LSC.

**Figure 3:**
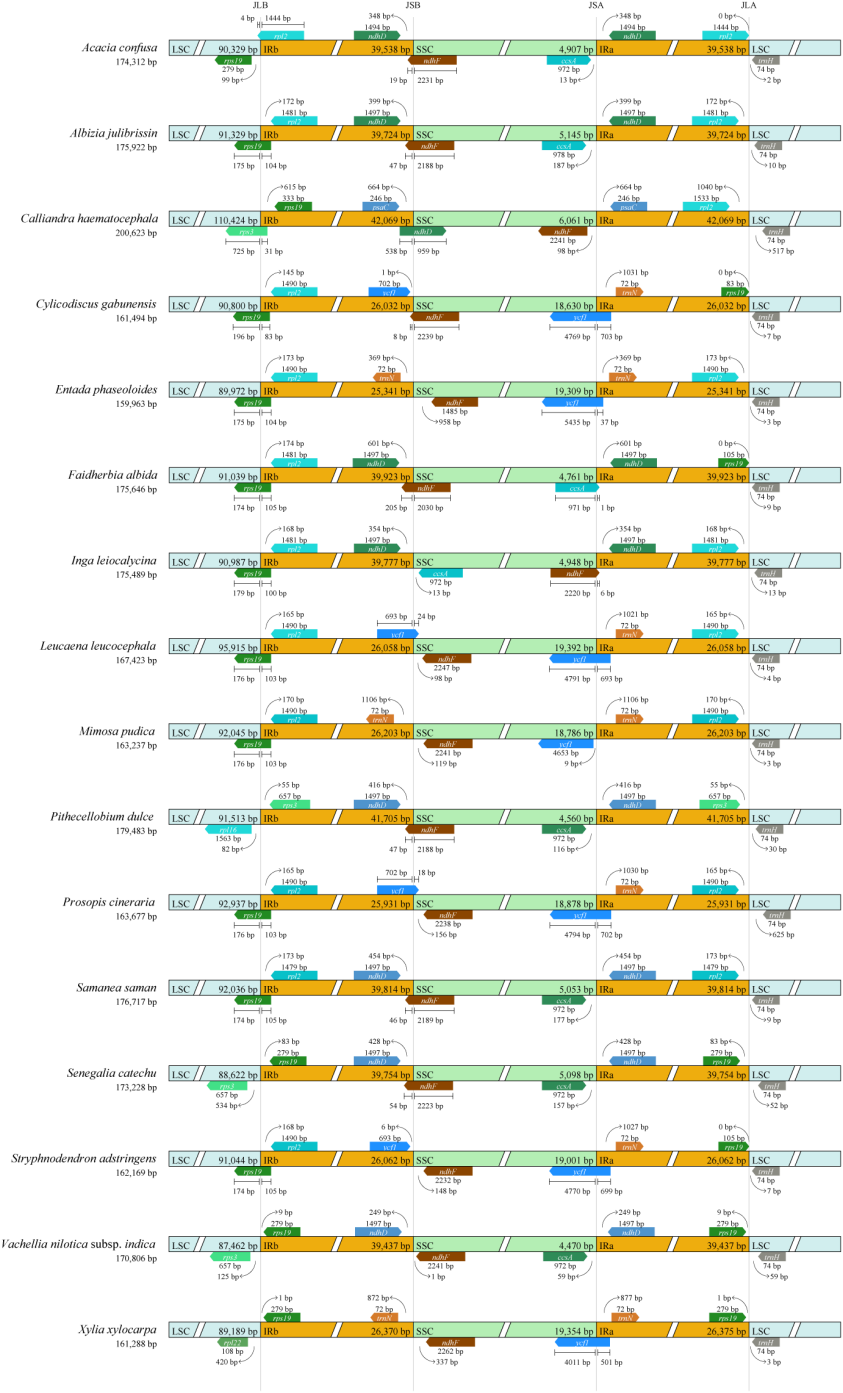
Comparison of the LSC, SSC, and IR boundaries of 16 plastomes from the tribe Mimoseae. JL_B_, JS_B_, JS_A_, and JL_A_ refer to the junctions of LSC/IRB, SSC/IRB, SSC/IRA, and LSC/IRA, respectively. The boxes above and below the line refer to the forward and reverse genes.

Three species [*E. phaseoloides*, *Mimosa pudica* L., and *Xylia xylocarpa* (Roxb.) W.Theob.] displayed IR contractions, with IR lengths ranging from 25,341 bp (*E. phaseoloides*) to 26,370 bp (*X. xylocarpa*), retaining entire *ndhF* in SSC. JSB of *E. phaseoloides*, *M. pudica*, and *X. xylocarpa* shifted between *trnN* and *ndhF*, while JSA remained within *ycf1* (*E. phaseoloides* and *X. xylocarpa*) or relocated between *ycf1* and *trnN* (*M. pudica*). The JLB of *E. phaseoloides* and *M. pudica* occurred within *rps19*, duplicating their 5’ ends (104 bp and 103 bp, respectively). *Xylia xylocarpa* exhibited a 0.2-kb IR expansion into LSC, positioning its JLB between *rpl22* and *rps19* and incorporating the entire *rps19* into the IR (**Figure 3**).

### 3.3 Identification of divergence hotspot regions

**Figure 4:**
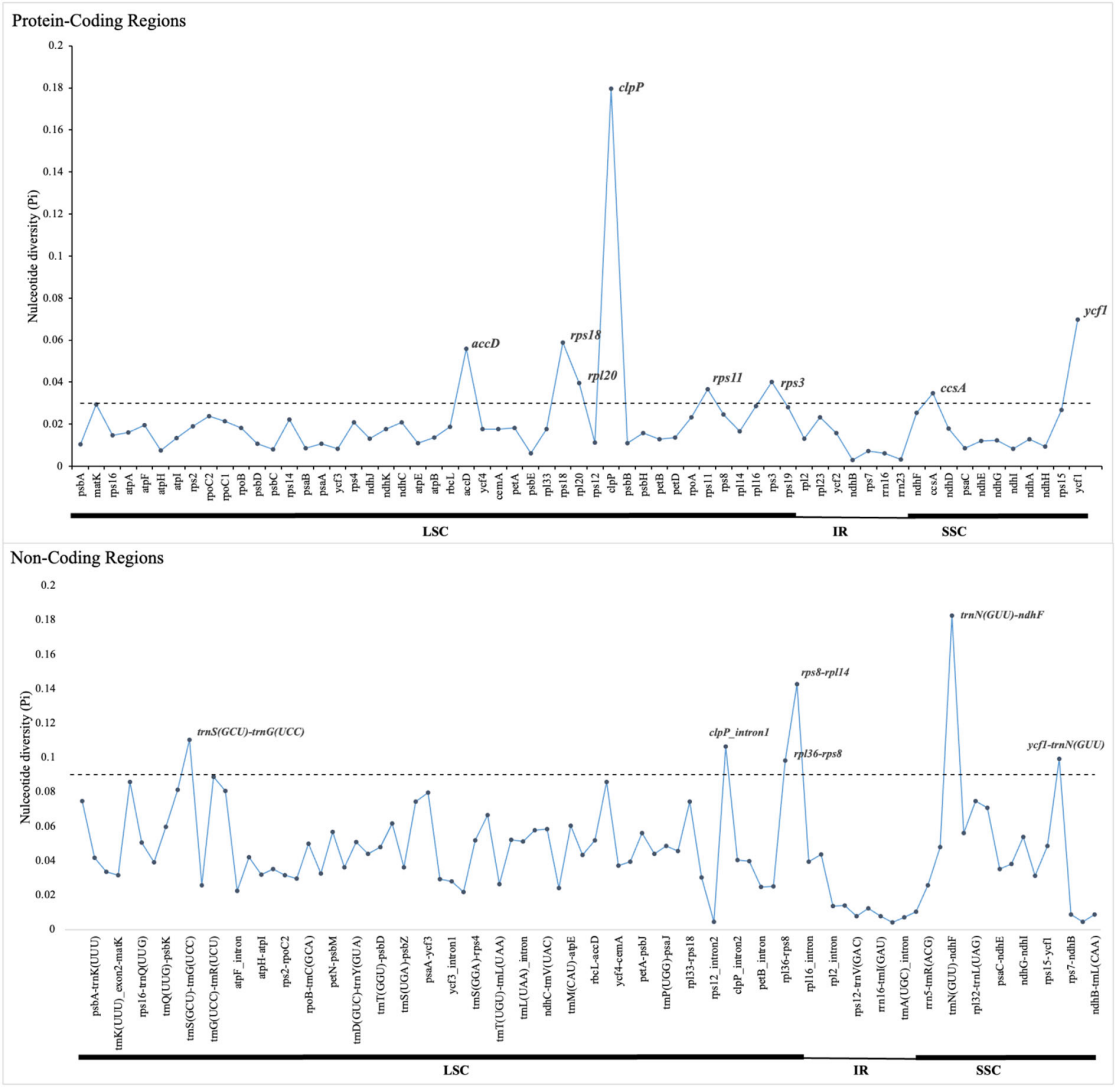
Nucleotide diversity (Pi) values of different regions of 16 plastomes from tribe Mimoseae. The dotted line indicates the threshold for high Pi values.

Comparative analysis of the 16 plastomes of Mimoseae revealed higher sequence variability in single copy regions than in IR regions, and in non-coding regions than in coding regions. Nucleotide diversity (Pi) analysis identified several highly variable regions (Pi > 0.03): *accD*, *rps18*, *rpl20*, *clpP*, *rps11*, and *rps3* (located in LSC), plus *ccsA* and *ycf1* (located in SSC). Among non-coding regions, *rps8*-*rpl14*, *trnS* (GCU)-*trnG* (UCC), *clpP*_intron1, and *rpl36*-*rps8* (located in LSC), as well as *trnN* (GUU)-*ndhF* and *ycf1*-*trnN* (GUU) (located in SSC) exhibited even higher nucleotide diversity (Pi > 0.09). Most other non-coding regions displayed Pi values between 0.03 and 0.09 (**Figure 4**).

### 3.4 Repeat analysis

**Figure 5:**
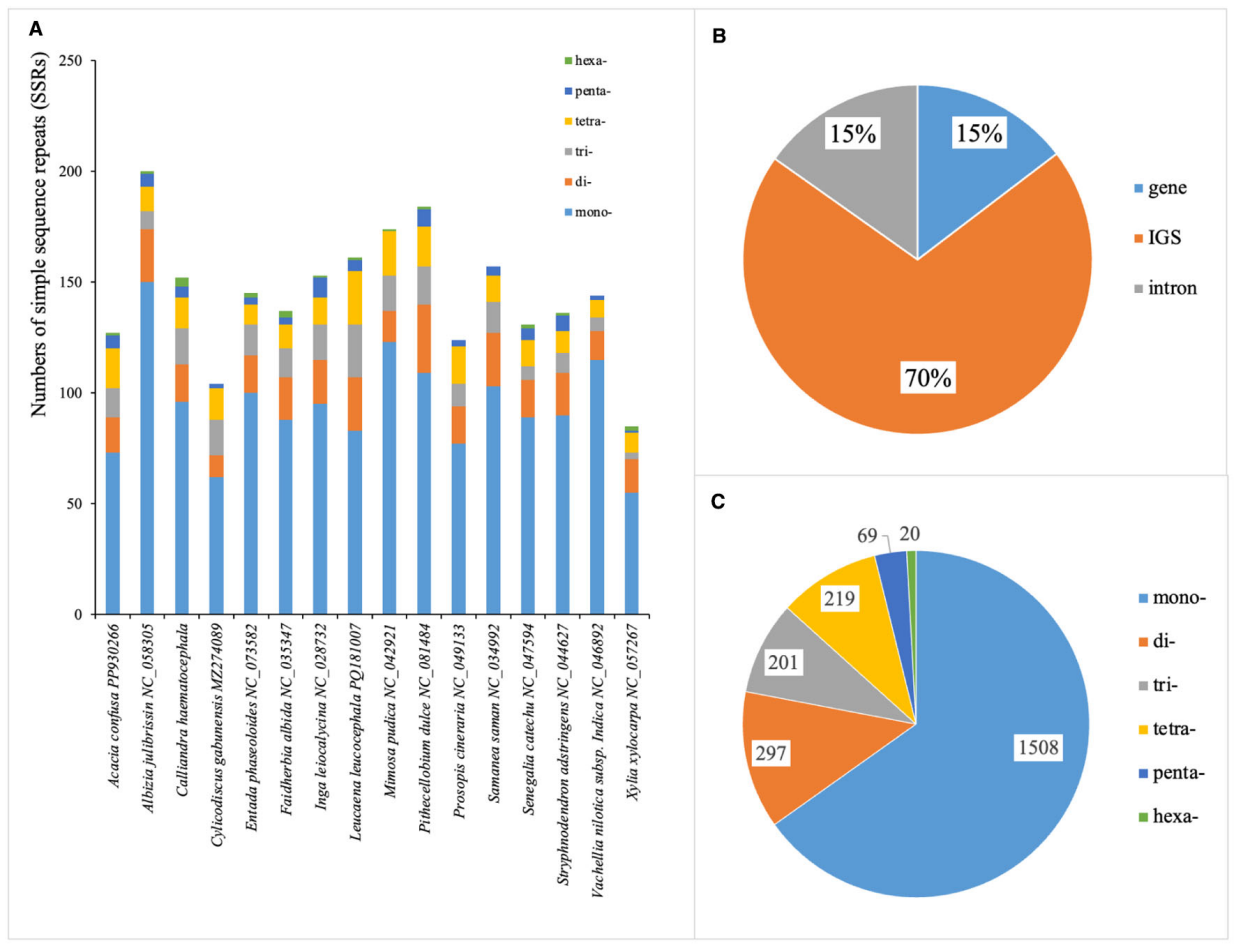
Distribution of simple sequence repeats (SSRs) in 16 plastomes of tribe Mimoseae. **(A)** Total numbers of SSRs detected in each species; **(B)** Distribution of SSRs in introns, coding sequences, and intergenic spacers (IGS); **(C)** Frequency of different SSR types.

A total of 2,314 SSRs were identified across the 16 plastomes of Mimoseae, ranging from 85 in *X. xylocarpa* to 200 in *A. julibrissin* (**Figure 5A**). The majority of these SSRs (~70%) were located in non-coding regions (**Figure 5B**). Among six types of SSR, mononucleotides constituted the largest proportion, followed by dinucleotides and tetranucleotides. Trinucleotides were less frequent, while pentanucleotides and hexanucleotides appeared only in a subset of plastomes (**Figure 5C**).

**Figure 6:**
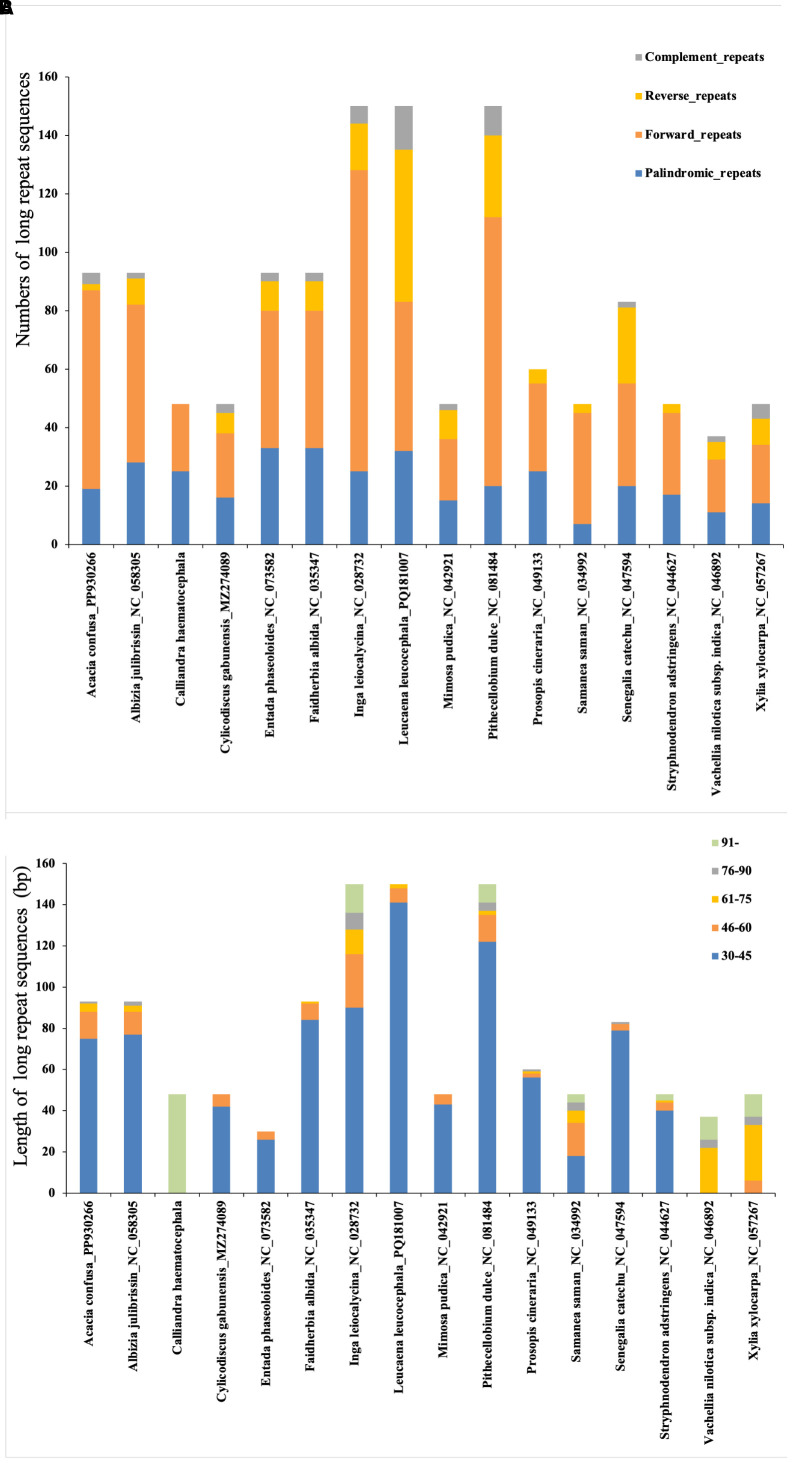
Analysis of long repeat sequences in 16 plastomes of tribe Mimoseae. **(A)** The types and abundance of long repeat sequences identified in each species; **(B)** Length distribution of these repeat sequences.

Additionally, we identified 1,290 long repeat sequences, comprising 697 forward, 196 reverse, 340 palindromic, and 57 complement repeats. The number of long repeats per plastome varied substantially, from 30 in *E. phaseoloides* to 150 in *P. dulce*, *L. leucocephala*, and *I. leiocalycina* (**Figure 6A**). Despite this interspecific variation, the distribution patterns of repeat types and length categories were largely conserved across species, with most repeats occurring in non-coding regions. Repeats of 30–45 bp predominated, followed by 45–60 bp, > 90 bp, 60–75 bp, and 75–90 bp (**Figure 6B**).

Notably, *C. haematocephala* exhibited an exceptional pattern, with 48 long repeat sequences identified. These included 25 palindromic and 23 forward repeats, while no reverse or complement repeats were detected. The lengths of these repeats ranged from 228 to 1,610 bp, all exceeding 90 bp.

### 3.5 Codon usage analyses

**Figure 7:**
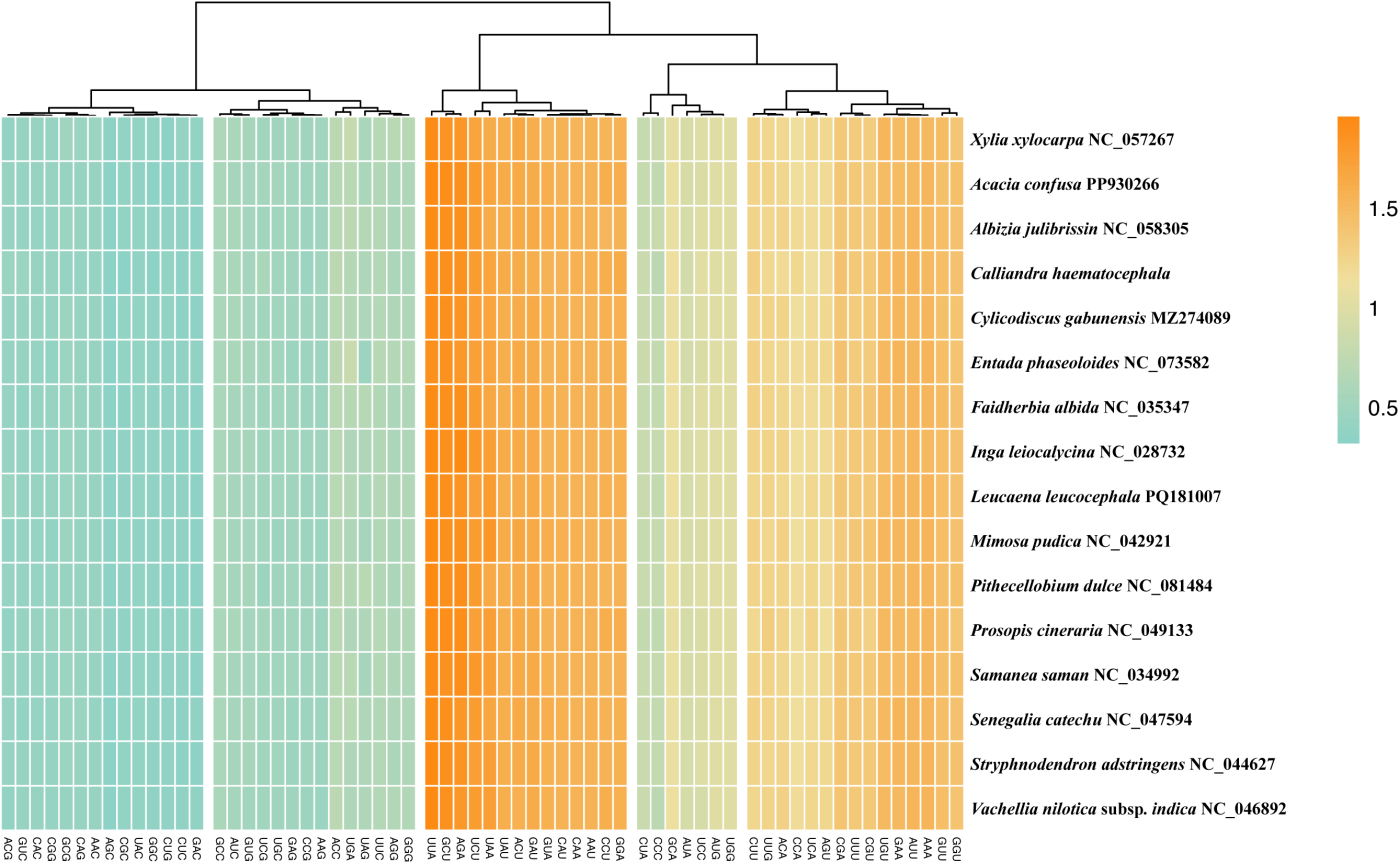
Relative synonymous codon usage (RSCU) analysis for protein-coding genes across 16 plastomes of the tribe Mimoseae.

Protein-coding regions in the 16 plastomes of Mimoseae contained 22,226 to 22,697 codons. AUU (encoding Isoleucine) was the most frequent codon (980 – 1,026 occurrences). UUA showed the highest mean relative synonymous codon usage (RSCU) value (mean value = 1.93). Thirty codons exhibited RSCU > 1, with 29 ending with A/U. Two codons, AUG (methionine) and UGG (tryptophan), had RSCU values of 1 in all 16 species, indicating no codon bias (**Figure 7**).

### 3.6 Phylogenetic inferences

**Figure 8:**
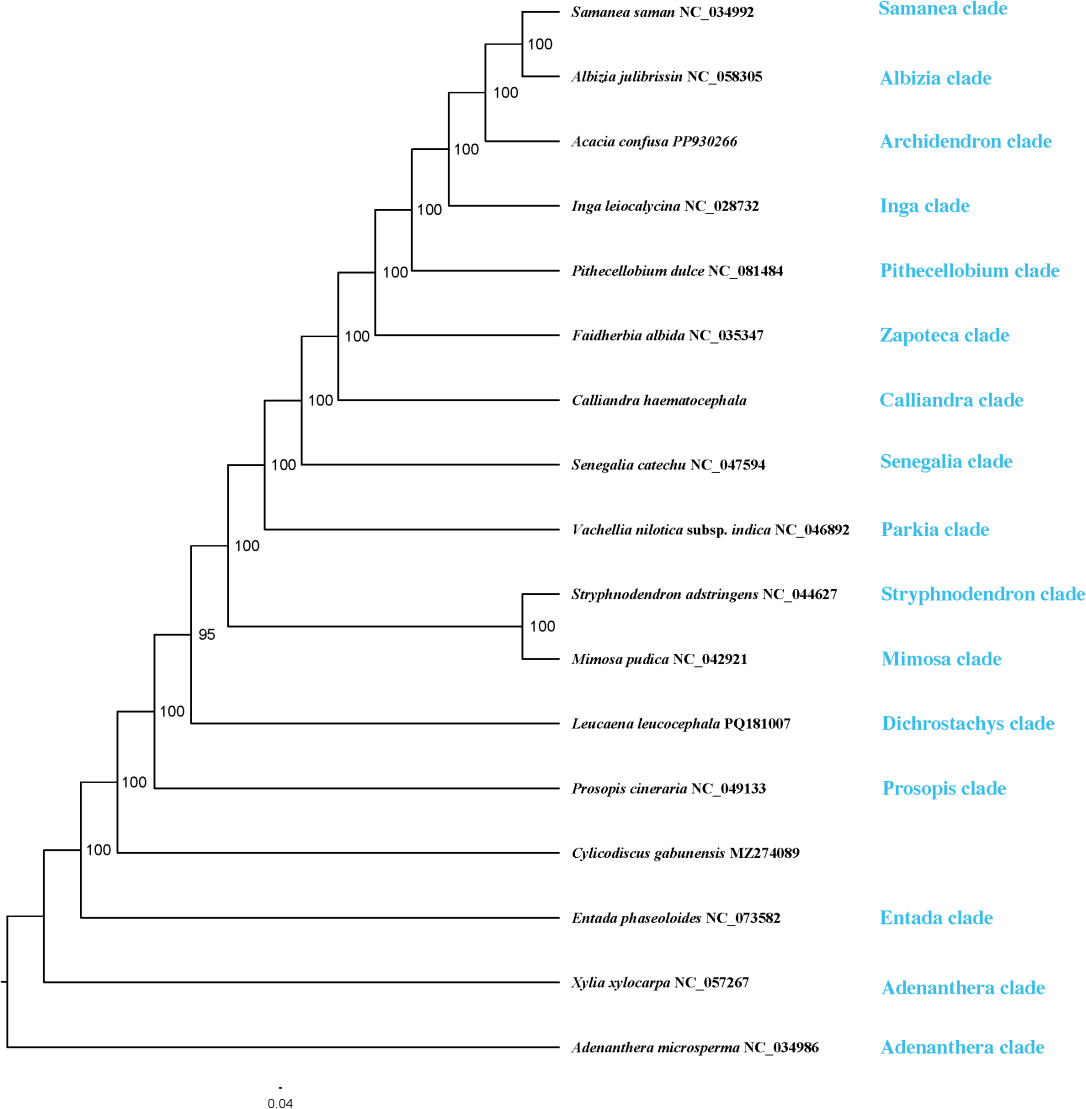
Maximum likelihood (ML) tree based on whole plastome sequences of 16 species of the tribe Mimoseae and one outgroup. Bootstrap values are shown at the branch nodes.

Phylogenetic trees reconstructed from the PCGs and CP matrices showed congruent topologies, with strong nodal support (bootstrap support, BS > 90%). The ML tree reconstructed from the whole plastome alignment (**Figure 8**) exhibited higher overall support, and displayed a topology largely congruent with previous studies (Bruneau et al., 2024; Queiroz et al., 2024), and served for subsequent selection pressure analyses.

### 3.7 Selection pressure analyses

The *dN/dS* ratios for 76 PCGs across the 16 Mimoseae plastomes ranged from 0.0001 to 2.04416. Three genes, *clpP*, *ycf2*, and *rps17*, showed *dN/dS* > 1, indicating positive selection, while *psaJ* and *psbI* exhibited *dN/dS* ratios close to 0 (0.0001).

**Figure 9:**
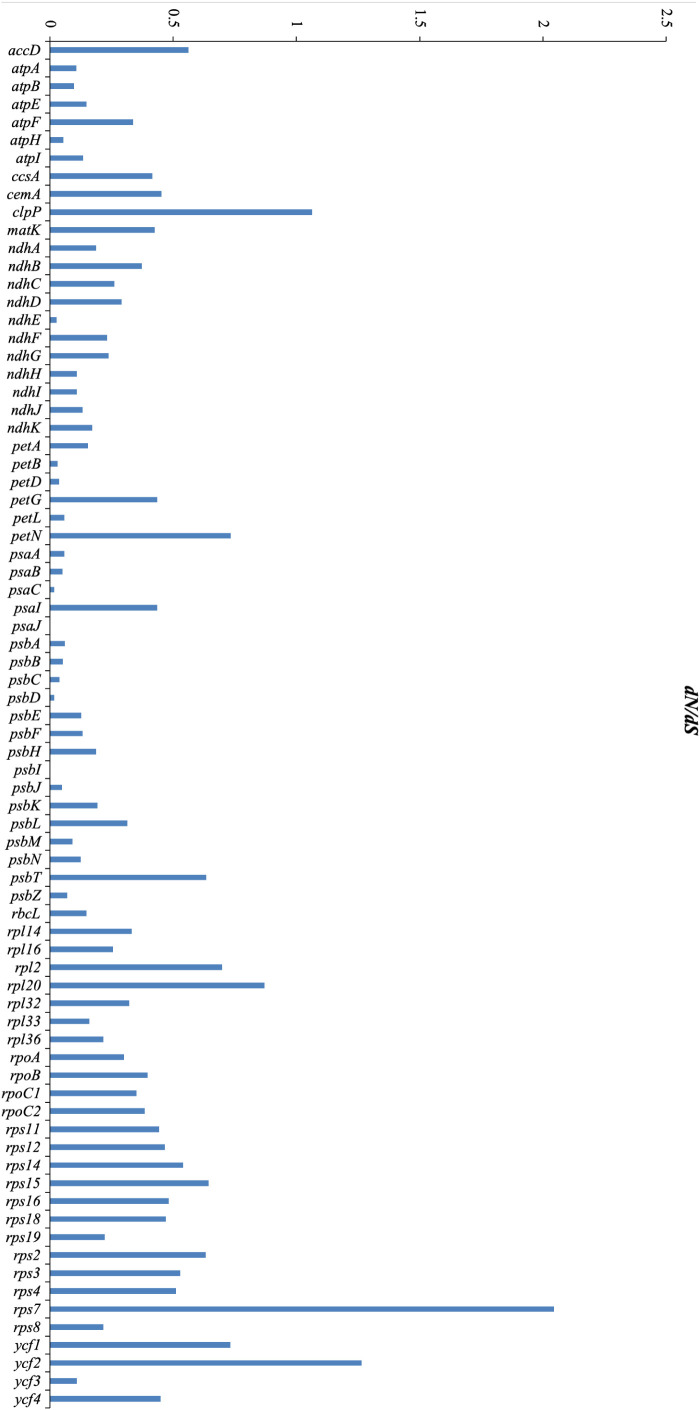
Distribution of non-synonymous/synonymous rate ratios (*dN*/*dS* ratios) for 76 protein-coding genes across 16 plastomes of the tribe Mimoseae.

Branch model analyses assessed differential selection between foreground and background branches. Likelihood ratio tests comparing M0 (one-ratio) and M2 (two-ratio) models favored M0 for most PCGs. After excluding abnormal *dN/dS* ratios (ratio = 999), 12 genes (*rpoC1*, *atpA*, *rpoB*, *rpoC2*, *rps11*, *rps12*, *atpB*, *clpP*, *rps18*, *rpoA*, *petB*, and *rpl14*) better fit M2 in the *Calliandra* lineage. Specifically, *rpoC1*, *atpA*, *rpoB*, *rps11*, *rps12*, *rps18*, and *rpl14* underwent positive selection; *rpoC2*, *rpoA*, and *petB* showed relaxed purifying selection; *atpB* experienced intensified purifying selection (**Figure 9**; **Supplementary Table S3**).

## 4 Discussion

### 4.1 Selection analysis indicating potential adaptative evolution

Positive selection (*dN/dS* > 1) was detected in *clpP*, *ycf2*, and *rps17*. The gene *clpP* encodes the ClpP protease, which maintains organellar protein homeostasis and mediates environmental stress responses (Yu and Houry, 2007; Wicke et al., 2011; Hong et al., 2020), such as high temperature, drought, and salt stress (Deng et al., 2016). Given that most species of Mimoseae exhibit drought-tolerant traits, the positive selection observed in *clpP* may be related to their enhanced adaptability to arid conditions (Wicke et al., 2011; Deng et al., 2016). The protein encoded by *ycf2* localizes to chloroplast membranes and participates in membrane assembly and homeostasis (Xing et al., 2025). The positive selection of the *ycf2* gene indirectly indicates that the tribe Mimoseae has a more stable chloroplast structure, thus adapting to more complex environments (Xing et al., 2025). The species of Mimoseae occupy broad distribution ranges (Bruneau et al., 2024). These positively selected genes might be involved in unique environmental adaptations in Mimoseae.

When the Calliandra clade was designated as the foreground branch, positive selection was detected in *rpoC1*, *atpA*, *rpoB*, *rps11*, *rps12*, *rps18*, and *rpl14*. Functionally, *rpoC1* encodes the β-subunit of chloroplast RNA polymerase in plants and is a core component of the chloroplast gene transcription mechanism, regulating the transcription of chloroplast genes (Wu et al., 2016; Peng et al., 2020), *rps11*, *rps12*, *rps18*, and *rpl14* function in protein synthesis (Wicke et al., 2011), *atpA* functions in photosynthesis, and *rpoB* encodes the β-subunit of plastid-encoded RNA polymerase (PEP) (Wicke et al., 2011; Wu et al., 2024). Concurrently, *atpB* (involved in photosynthesis) exhibited intensified purifying selection, while *rpoC2*, *rpoA* (transcription), and *petB* (photosynthesis) displayed relaxed purifying selection. The primary role of *atpB* in photophosphorylation and energy homeostasis links it to abiotic stress responses (Li et al., 2010; Li et al., 2022). These selected genes are mainly related to chloroplast function including photosynthesis and may enhance the plant’s environmental adaptability by regulating photosynthetic efficiency (Wu et al., 2016; Peng et al., 2020; Wu et al., 2024). These genes likely experienced distinct evolutionary histories. In addition, our study found higher nucleotide diversity in non-coding regions than in coding regions, consistent with most previous studies (Wang, 2017; Contreras-Díaz et al., 2021). These identified hypervariable regions (both coding and non-coding) may complement previous studies and serve as valuable markers for phylogenetic, population genetic, and barcoding studies in Mimoseae or other plant taxa.

### 4.2 Significant structural variations indicating high plastome diversity in Mimoseae

The plastome of *C. haematocephala* exhibits significant structural features that highlight the plastomic diversity within Mimoseae. Most notably, it displays substantial plastome expansion and extensive structural rearrangements, closely associated with a ~14-kb expansion of the inverted repeat (IR) region into the large single-copy (LSC) region (Chumley et al., 2006). This IR expansion results in exceptionally large IRs (42,069 bp), contributing to a total plastome size of 200,623 bp—the largest reported to date within Mimoseae and across Leguminosae.

A prominent feature of this plastome is the high abundance of clustered dispersed repeats, ranging from 228 to 1,610 bp. These repeats likely serve as structural hotspots, promoting duplications, inversions, and the accumulation of additional repeat elements (Jin, 2019). Geneious-based (Kearse et al., 2012) visualization revealed that these repeats are non-randomly distributed and often clustered, potentially mediating large-scale rearrangements through intramolecular recombination.

Assembly of the *C. haematocephala* plastome posed challenges using short-read sequencing alone, resulting in a short 23-kb contig or fragmented contigs due to unresolved long repeats. In contrast, PacBio HiFi long reads enabled complete circular assembly using tools such as Oatk (Zhou et al., 2024) and TIPPo (Xian et al., 2025), confirming the accuracy and necessity of long-read sequencing for plastomes with complex repetitive structures. We recommend this approach for plastomes that cannot be circularized using short-read sequencing alone.

Collinearity analysis revealed extensive plastomic rearrangements in *C. haematocephala*. Although IR loss in the IRLC of Papilionoideae has been inferred to be associated with structural rearrangements (Palmer and Thompson, 1982; Wicke et al., 2011), some IR-lacking species (e.g., *Medicago sativa* L. and *Wisteria floribunda* (Willd.) DC.) exhibit limited structural variation (Wolfe et al., 1987; Lee et al., 2021). Conversely, substantial rearrangements have been observed in multiple IR-retaining lineages, including Campanulaceae (Wu and Chaw, 2014), Oleaceae (Cosner et al., 2004), Plantaginaceae (Zhu et al., 2016), and *Pelargonium* (Lee et al., 2007). Studies on *Erodium* further confirmed that plastome stability lacks a direct correlation with IR presence (Blazier et al., 2016; Wang et al., 2022). The extensive rearrangements observed in IR-retaining *C. haematocephala* are consistent with these findings.

In Mimosoideae, Wang (2017) revealed an approximately 13-kb IR expansion into the SSC region in the clade formed by tribes Ingeae and *Acacia* s.s (Dugas et al., 2015; Williams et al., 2015). Notably, *C. haematocephala* exhibited an additional 0.7-kb IR expansion into the LSC, shifting the JLB boundary into *rps3*. This IR expansion contributes to its exceptionally large plastome and further elucidates IR dynamics in Mimoseae. Phylogenetically, *C. haematocephala* belongs to the Calliandra clade, distinct from the Senegalia grade and the Zapoteca clade (Bruneau et al., 2024). Broader sampling of *Calliandra* plastomes is needed to assess whether IR expansion is characteristic of this lineage.

In comparison to previously reported plastome sizes in Leguminosae in GenBank such as *Faidherbia albida* (175,675 bp) (Mensous et al., 2017), and recently, Zhang et al. (2025) assembled 235 plastomes, with the largest circularized plastome being *Pseudosamanea guachapele* (Kunth) Harms (182,795 bp), our assembly of *C. haematocephala* (200,623 bp) now represents the largest plastome reported in Leguminosae. Elevated dispersed repeat abundance is known to drive plastomic rearrangements (Guisinger et al., 2008; Guisinger et al., 2011; Xu et al., 2023), and likely underlies the extensive structural variations in *C. haematocephala*. As in other Mimoseae species (Dugas et al., 2015; Wang, 2017), the predominant dispersed repeats in *C. haematocephala* are palindromic and forward repeats. Similarly, in Geraniaceae, plastome variations in *Pelargonium* × *hortorum* L.H.Bailey involved at least 12 inversions (Chumley et al., 2006). Weng et al. (2014) further showed that inversions were a major driver of plastome variability in 11 Geraniaceae species, with large insertions positively correlated with structural variation and distribution of repeat sequences strongly associated with breakpoints (Chumley et al., 2006). Previous researches demonstrated that plastome rearrangements in the Putranjivoids clade (Malpighialean) correlated with the abundance of repeat sequences (Wolfe et al., 1987; Jin et al., 2019).

Molecular mechanisms underlying plastome structural changes include repeat-mediated homologous recombination, slipped-strand mispairing, and occasional foreign-DNA integration (Cauz-Santos, 2025). Several studies have proposed that dispersed repeats (Ogihara et al., 1988; Wang et al., 2024; Cauz-Santos, 2025), particularly long forward repeats, are key mediators of inversions and rearrangements. For example, Wang et al. (2022) demonstrated that both the number and length of dispersed repeats are positively correlated with rearrangement frequency and magnitude. In *C. haematocephala*, all forward repeats exceed 90 bp, suggesting their active role in recombination-mediated plastome remodeling. IR dynamics are also recognized as major drivers of plastome structural evolution (Cauz-Santos, 2025). Shifts in IR boundaries through recombination or gene conversion can lead to expansions, contractions, or even gene duplication. A well-documented case in *Nicotiana acuminata* (Graham) Hook. involved a >12-kb IR expansion triggered by a double-strand break and recombination event (Goulding et al., 1996). In *N. tabacum*, experimental removal of the IR region led to altered gene dosage and increased plastome copy number (Krämer et al., 2024), emphasizing the IR’s role in plastome architecture and regulation.

In *C. haematocephala*, the 14-kb IR expansion may be associated with insertions, deletions, or duplications of genes near IR junctions. These changes, in combination with abundant dispersed repeats, likely contribute to the extensive plastomic rearrangements observed in this species. Notably, these findings support a growing consensus that plastome size and structure are determined by the interplay between repeat content and IR boundary dynamics, rather than IR presence or absence alone.

## 5 Conclusion

The plastome of *C. haematocephala* is remarkable in both structure and size. We found that it possesses the largest reported plastome in Mimoseae, and indeed within Leguminosae to date. The large increase in size and the high number of rearrangements are associated with a series of major IR expansions, as well as translocations and inversions. Furthermore, an abundance of clustered dispersed repeats has also been identified as a key factor contributing to the extensive plastomic rearrangements and the increase in plastome size. Selection pressure analyses identified positive selection in *clpP*, *ycf2*, and *rps17*, suggesting their potential roles in adaptive evolution. Branch-specific positive selection was also detected in genes such as *rpoC1* and *atpA* within the Calliandra clade, indicating lineage-specific adaptive pressures.

## Data Availability

The original contributions presented in the study are publicly available. The newly sequenced plastome has been deposited in GenBank under accession number PX367310 (**Supplementary Table S4**). The supplementary tables of this study and data matrix used for phylogenetic analysis are available at Figshare (doi.org/10.6084/m9.figshare.30050251).
