# Supplementary material for: Dispersed repeats and inverted repeat expansion drive major plastomic rearrangements in Calliandra haematocephala (Leguminosae: Mimoseae)
Source: Front Plant Sci. 2025 Oct 3;16:1673127. doi: 10.3389/fpls.2025.1673127 (PMC12531236; doi:10.3389/fpls.2025.1673127)
Supplement: Supplementary file 2 [file DataSheet2.zip › Physical maps of plastomes in this study/Albizia_julibrissin_NC_058305.gb.pdf]

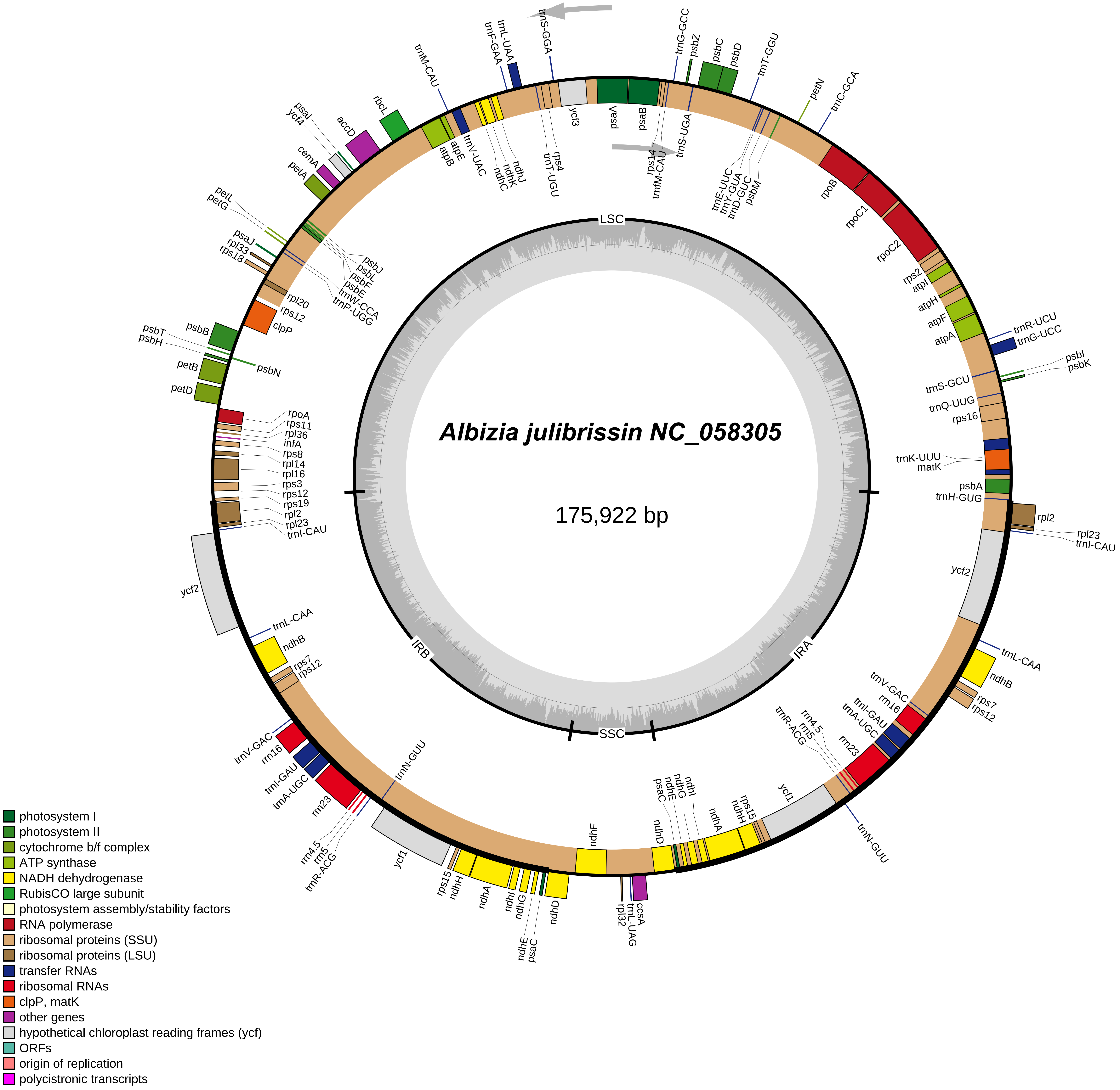

Albizia julibrissin NC_058305
175,922 bp
LSC
SSC
IRA
IRB
trnS-GGA
trnL-UAA
trnF-GAA
trnM-CAU
rbcL
accD
psaI
ycf4
cemA
petA
petL
petG
psaJ
rpl33
rps18
rpl20
rps12
clpP
psbB
psbT
psbH
psbN
petB
petD
rpoA
rps11
rpl36
infA
rps8
rpl14
rpl16
rps3
rps12
rps19
rpl2
rpl23
trnI-CAU
ycf2
trnL-CAA
ndhB
rps7
rps12
trnV-GAC
rrn16
trnI-GAU
trnA-UGC
rrn23
rrn4.5
rrn5
trnR-ACG
trnN-GUU
ycf1
rps15
ndhH
ndhA
ndhI
ndhG
ndhE
psaC
ndhD
ndhF
rpl32
trnL-UAG
ccsA
ndhD
psaC
ndhE
ndhG
ndhI
ndhA
ndhH
rps15
ycf1
trnN-GUU
trnR-ACG
rrn5
rrn4.5
rrn23
trnA-UGC
trnI-GAU
rrn16
trnV-GAC
rps12
rps7
ndhB
trnL-CAA
ycf2
trnI-CAU
rpl23
rpl2
trnH-GUG
psbA
matK
trnK-UUU
rps16
trnQ-UUG
trnS-GCU
psbK
psbI
trnG-UCC
trnR-UCU
atpA
atpF
atpH
atpI
rps2
rpoC2
rpoC1
rpoB
trnC-GCA
petN
psbM
trnD-GUC
trnY-GUA
trnE-UUC
trnT-GGU
psbD
psbC
psbZ
trnG-GCC
trnS-UGA
trnfM-CAU
rps14
psaB
psaA
ycf3
rps4
trnT-UGU
ndhJ
ndhK
ndhC
trnV-UAC
atpE
atpB
psbJ
psbL
psbF
psbE
trnW-CCA
trnP-UGG
photosystem I
photosystem II
cytochrome b/f complex
ATP synthase
NADH dehydrogenase
RubisCO large subunit
photosystem assembly/stability factors
RNA polymerase
ribosomal proteins (SSU)
ribosomal proteins (LSU)
transfer RNAs
ribosomal RNAs
clpP, matK
other genes
hypothetical chloroplast reading frames (ycf)
ORFs
origin of replication
polycistronic transcripts
