# Supplementary material for: Dispersed repeats and inverted repeat expansion drive major plastomic rearrangements in Calliandra haematocephala (Leguminosae: Mimoseae)
Source: Front Plant Sci. 2025 Oct 3;16:1673127. doi: 10.3389/fpls.2025.1673127 (PMC12531236; doi:10.3389/fpls.2025.1673127)
Supplement: Supplementary file 2 [file DataSheet2.zip › Physical maps of plastomes in this study/Calliandra_haematocephala.gb.pdf]

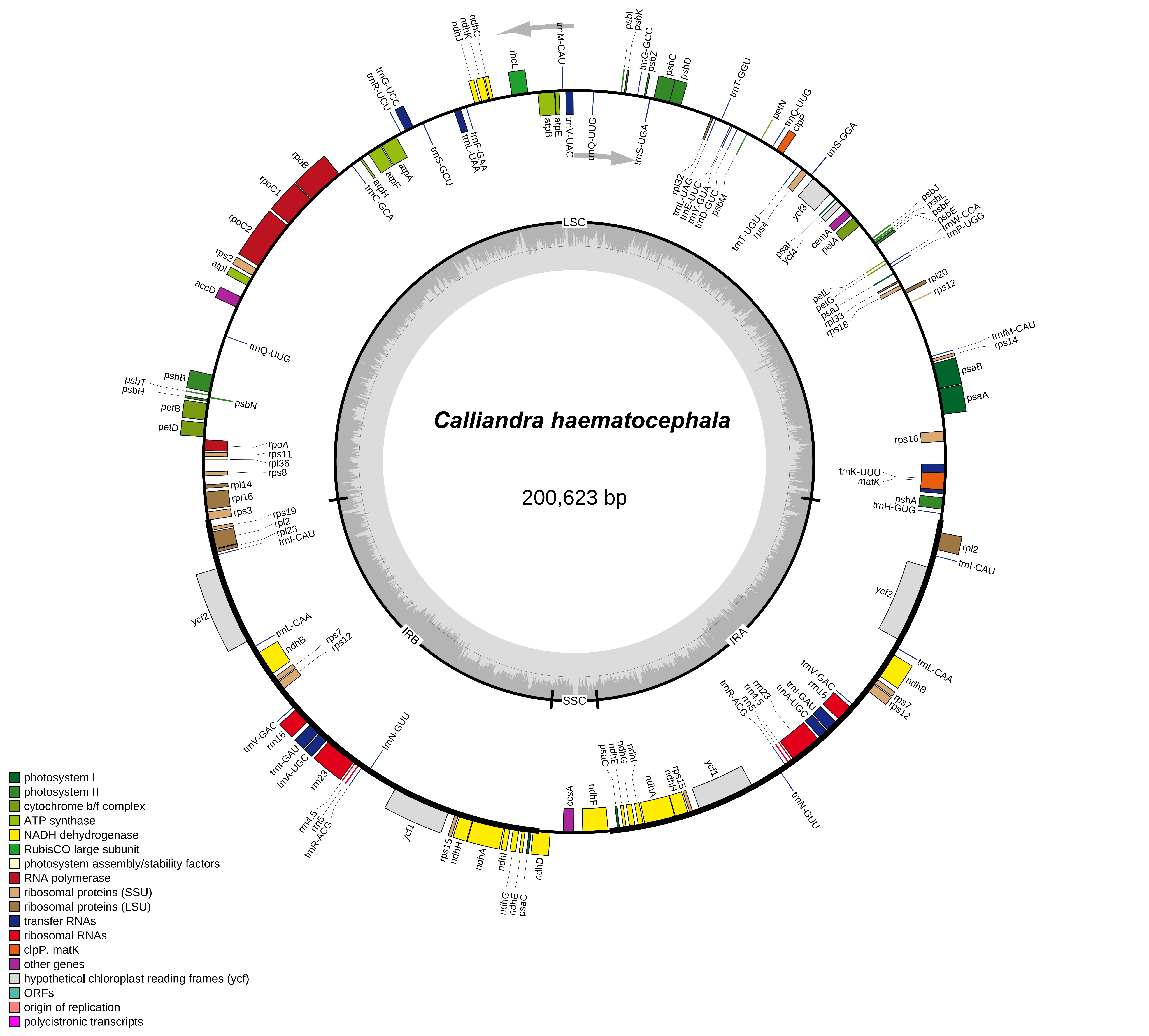

Calliandra haematocephala
200,623 bp
LSC
IRA
SSC
IRB
photosystem I
photosystem II
cytochrome b/f complex
ATP synthase
NADH dehydrogenase
RubisCO large subunit
photosystem assembly/stability factors
RNA polymerase
ribosomal proteins (SSU)
ribosomal proteins (LSU)
transfer RNAs
ribosomal RNAs
clpP, matK
other genes
hypothetical chloroplast reading frames (ycf)
ORFs
origin of replication
polycistronic transcripts
trnM-CAU
rbcL
ndhC
ndhK
ndhJ
atpE
atpB
trnV-UAC
trnQ-UUG
psbI
psbK
trnG-GCC
psbZ
psbC
psbD
trnS-UGA
trnT-GGU
petN
trnQ-UUG
clpP
trnS-GGA
rpl32
trnL-UAG
trnE-UUC
trnY-GUA
trnD-GUC
psbM
trnT-UGU
rps4
ycf3
psaI
ycf4
cemA
petA
psbJ
psbL
psbF
psbE
trnW-CCA
trnP-UGG
rpl20
rps12
petL
petG
psaJ
rpl33
rps18
trnfM-CAU
rps14
psaB
psaA
rps16
trnK-UUU
matK
psbA
trnH-GUG
rpl2
trnI-CAU
ycf2
trnL-CAA
ndhB
rps7
rps12
trnV-GAC
rrn16
trnI-GAU
trnA-UGC
rrn23
rrn4.5
rrn5
trnR-ACG
trnN-GUU
ycf1
rps15
ndhH
ndhA
ndhI
ndhG
ndhE
psaC
ndhD
ndhF
ccsA
trnG-UCC
trnR-UCU
trnF-GAA
trnL-UAA
trnS-GCU
atpA
atpF
atpH
trnC-GCA
rpoB
rpoC1
rpoC2
rps2
atpI
accD
trnQ-UUG
psbB
psbT
psbH
psbN
petB
petD
rpoA
rps11
rpl36
rps8
rpl14
rpl16
rps3
rps19
rpl2
rpl23
trnI-CAU
