# Supplementary material for: Dispersed repeats and inverted repeat expansion drive major plastomic rearrangements in Calliandra haematocephala (Leguminosae: Mimoseae)
Source: Front Plant Sci. 2025 Oct 3;16:1673127. doi: 10.3389/fpls.2025.1673127 (PMC12531236; doi:10.3389/fpls.2025.1673127)
Supplement: Supplementary file 2 [file DataSheet2.zip › Physical maps of plastomes in this study/Faidherbia_albida_NC_035347.gb.pdf]

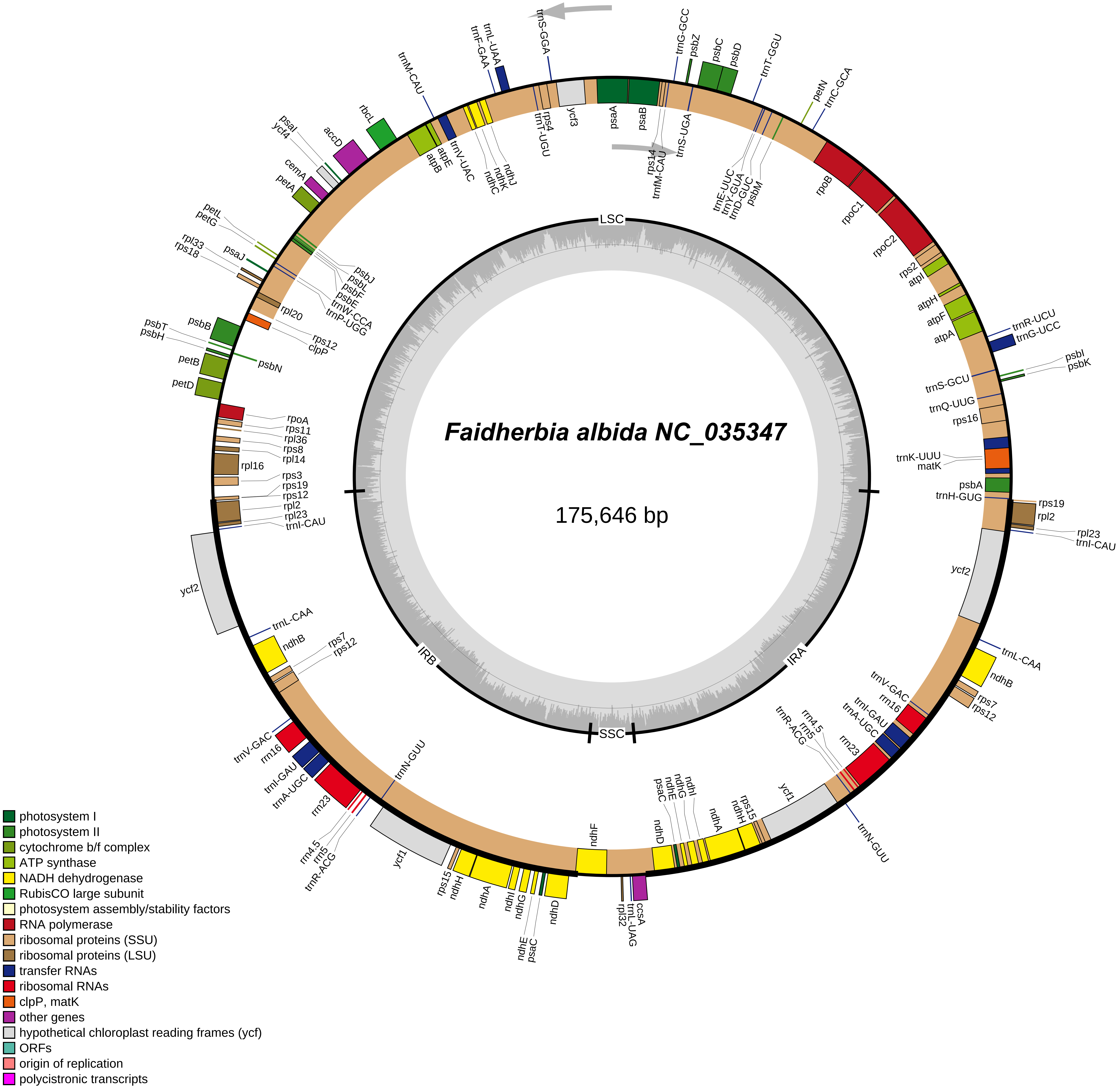

Faidherbia albida NC_035347
175,646 bp
LSC
IRA
SSC
IRB
trnS-GGA
trnL-UAA
trnF-GAA
trnM-CAU
rbcL
accD
psaI
ycf4
cemA
petA
petL
petG
rpl33
rps18
psaJ
psbT
psbH
psbB
petB
petD
psbN
rpl20
rps12
clpP
psbJ
psbL
psbF
psbE
trnW-CCA
trnP-UGG
atpE
atpB
trnV-UAC
ndhC
ndhK
ndhJ
rps4
trnT-UGU
ycf3
psaA
psaB
rps14
trnfM-CAU
trnS-UGA
trnG-GCC
psbZ
psbC
psbD
trnT-GGU
trnE-UUC
trnY-GUA
trnD-GUC
psbM
petN
trnC-GCA
rpoB
rpoC1
rpoC2
rps2
atpI
atpH
atpF
atpA
trnR-UCU
trnG-UCC
psbI
psbK
trnS-GCU
trnQ-UUG
rps16
trnK-UUU
matK
psbA
trnH-GUG
rps19
rpl2
rpl23
trnI-CAU
ycf2
trnL-CAA
ndhB
rps7
rps12
trnV-GAC
rrn16
trnI-GAU
trnA-UGC
rrn23
rrn4.5
rrn5
trnR-ACG
trnN-GUU
ycf1
rps15
ndhH
ndhA
ndhI
ndhG
ndhE
psaC
ndhD
ndhF
rpl32
trnL-UAG
ccsA
rpoA
rps11
rpl36
rps8
rpl14
rpl16
rps3
rps19
rps12
rpl2
rpl23
trnI-CAU
ycf2
trnL-CAA
ndhB
rps7
rps12
trnV-GAC
rrn16
trnI-GAU
trnA-UGC
rrn23
rrn4.5
rrn5
trnR-ACG
trnN-GUU
ycf1
photosystem I
photosystem II
cytochrome b/f complex
ATP synthase
NADH dehydrogenase
RubisCO large subunit
photosystem assembly/stability factors
RNA polymerase
ribosomal proteins (SSU)
ribosomal proteins (LSU)
transfer RNAs
ribosomal RNAs
clpP, matK
other genes
hypothetical chloroplast reading frames (ycf)
ORFs
origin of replication
polycistronic transcripts
