# Supplementary material for: Dispersed repeats and inverted repeat expansion drive major plastomic rearrangements in Calliandra haematocephala (Leguminosae: Mimoseae)
Source: Front Plant Sci. 2025 Oct 3;16:1673127. doi: 10.3389/fpls.2025.1673127 (PMC12531236; doi:10.3389/fpls.2025.1673127)
Supplement: Supplementary file 2 [file DataSheet2.zip › Physical maps of plastomes in this study/Xylia_xylocarpa_NC_057267.gb.pdf]

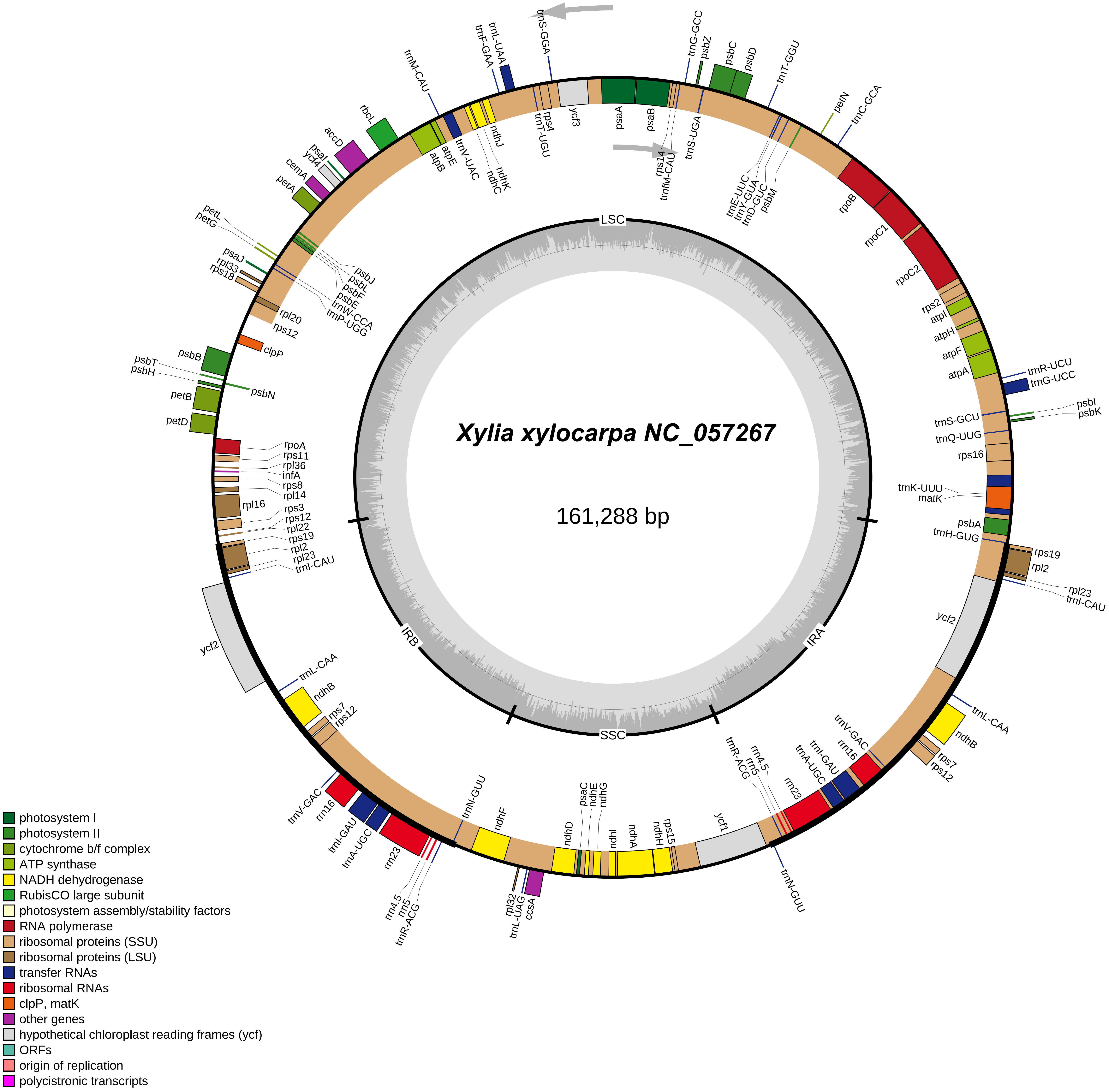
Xylia xylocarpa NC_057267
161,288 bp
LSC
IRA
IRB
SSC
psaA
psaB
psbC
psbD
rbcL
accD
ycf3
ycf2
ycf1
rpoB
rpoC1
rpoC2
ndhB
ndhF
ndhA
rrn23
rrn16
psbB
petB
petD
rpoA
rpl16
rpl2
matK
psbA
clpP
photosystem I
photosystem II
cytochrome b/f complex
ATP synthase
NADH dehydrogenase
RubisCO large subunit
photosystem assembly/stability factors
RNA polymerase
ribosomal proteins (SSU)
ribosomal proteins (LSU)
transfer RNAs
ribosomal RNAs
clpP, matK
other genes
hypothetical chloroplast reading frames (ycf)
ORFs
origin of replication
polycistronic transcripts
