# Supplementary figures and images for: Dispersed repeats and inverted repeat expansion drive major plastomic rearrangements in Calliandra haematocephala (Leguminosae: Mimoseae)
Source: Front Plant Sci. 2025 Oct 3;16:1673127. doi: 10.3389/fpls.2025.1673127 (PMC12531236; doi:10.3389/fpls.2025.1673127)

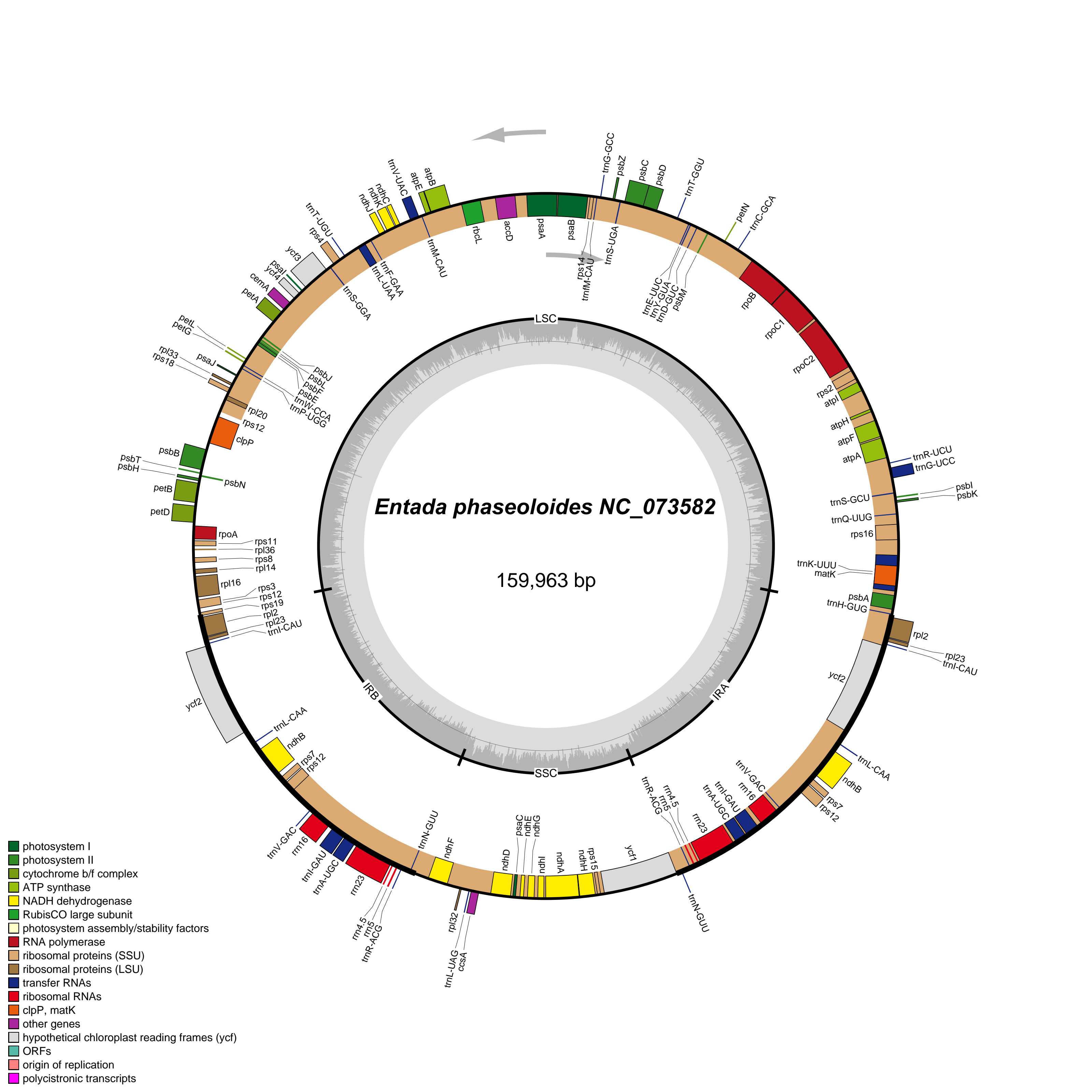

Supplement: Supplementary file 2 [file DataSheet2.zip › Physical maps of plastomes in this study/Entada_phaseoloides_NC_073582.gb.pdf]
