# Supplementary material for: Dispersed repeats and inverted repeat expansion drive major plastomic rearrangements in Calliandra haematocephala (Leguminosae: Mimoseae)
Source: Front Plant Sci. 2025 Oct 3;16:1673127. doi: 10.3389/fpls.2025.1673127 (PMC12531236; doi:10.3389/fpls.2025.1673127)
Supplement: Supplementary file 2 [file Acacia_confusa_PP930266.gb.pdf › Physical maps of plastomes in this study/Acacia_confusa_PP930266.gb.pdf]

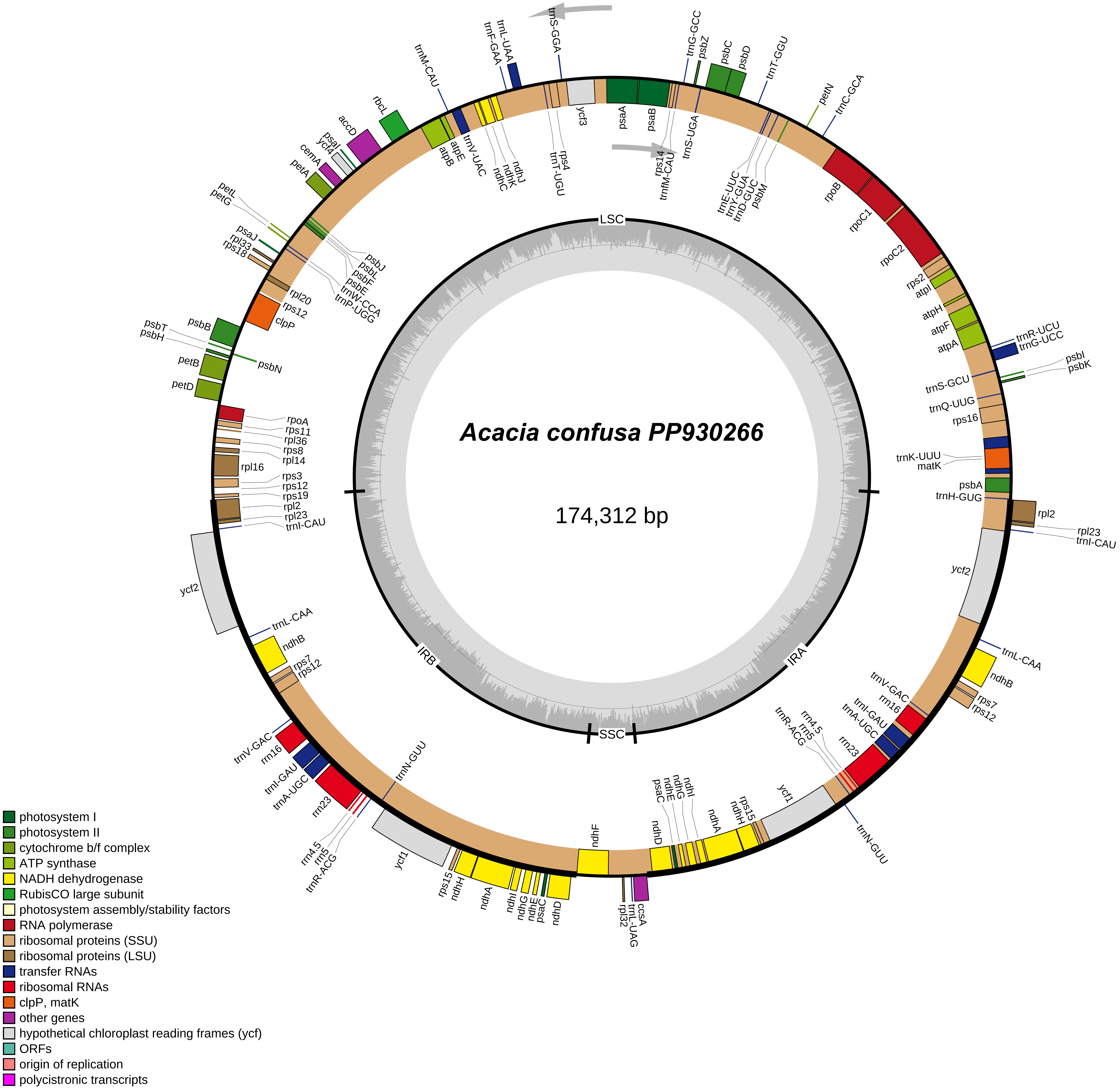
Acacia confusa PP930266
174,312 bp
LSC
IRA
SSC
IRB
trnS-GGA
trnL-UAA
trnF-GAA
trnM-CAU
rbcL
accD
psaI
ycf4
cemA
petA
petL
petG
psaJ
rpl33
rps18
rpl20
rps12
clpP
psbB
psbT
psbH
psbN
petB
petD
rpoA
rps11
rpl36
rps8
rpl14
rpl16
rps3
rps12
rps19
rpl2
rpl23
trnI-CAU
ycf2
trnL-CAA
ndhB
rps7
rps12
trnV-GAC
rrn16
trnI-GAU
trnA-UGC
rrn23
rrn4.5
rrn5
trnR-ACG
trnN-GUU
ycf1
rps15
ndhH
ndhA
ndhI
ndhG
ndhE
psaC
ndhD
ndhF
rpl32
trnL-UAG
ccsA
psbJ
psbL
psbF
psbE
trnW-CCA
trnP-UGG
atpE
atpB
trnV-UAC
ndhC
ndhK
ndhJ
trnT-UGU
rps4
ycf3
psaA
psaB
rps14
trnfM-CAU
trnS-UGA
trnG-GCC
psbZ
psbC
psbD
trnT-GGU
petN
trnC-GCA
trnE-UUC
trnY-GUA
trnD-GUC
psbM
rpoB
rpoC1
rpoC2
rps2
atpI
atpH
atpF
atpA
trnR-UCU
trnG-UCC
psbI
psbK
trnS-GCU
trnQ-UUG
rps16
trnK-UUU
matK
psbA
trnH-GUG
photosystem I
photosystem II
cytochrome b/f complex
ATP synthase
NADH dehydrogenase
RubisCO large subunit
photosystem assembly/stability factors
RNA polymerase
ribosomal proteins (SSU)
ribosomal proteins (LSU)
transfer RNAs
ribosomal RNAs
clpP, matK
other genes
hypothetical chloroplast reading frames (ycf)
ORFs
origin of replication
polycistronic transcripts
